# The maternal reduced uteroplacental perfusion model of preeclampsia induces sexually dimorphic metabolic responses in rat offspring

**DOI:** 10.1186/s13293-022-00458-8

**Published:** 2022-09-15

**Authors:** Mohammadmehdi Hassanzadeh-Taheri, Mahtab Mohammadifard, Zahra Erfanian, Mehran Hosseini

**Affiliations:** 1 Cellular and Molecular Research Center, Department of Anatomical Sciences, Birjand University of Medical Sciences, Birjand, Iran; 2 Department of Pathology, Faculty of Medicine, Birjand University of Medical Sciences, Birjand, Iran; 3 Faculty of Medicine, Birjand University of Medical Sciences, Birjand, Iran

**Keywords:** Preeclampsia, Offspring, Growth disorders, Metabolic dysfunction, Diabetes, Ghrelin

## Abstract

**Background:**

Offspring born to preeclamptic mothers are prone to obesity, diabetes and hypertension in later life, but still, studies investigating the underlying mechanism are limited. Here, we aimed to investigate the impact of the reduced uteroplacental perfusion (RUPP) rat preeclampsia model on offspring metabolic outcomes.

**Methods:**

Timed pregnant Wistar rats underwent RUPP or sham surgeries on day 14 of gestation. Glucometabolic parameters were evaluated on postnatal days (PND), 14 (childhood), and 60 (young adult). In addition, intraperitoneal glucose tolerance test (IPGTT), homeostatic model assessment of insulin resistance (HOMA-IR), immunohistochemical staining for insulin in pancreatic islets, arterial blood pressure and 24-h urine protein (24hUP) excretion were performed at PND60.

**Results:**

Male, but not female, young adult rats (PND60) of RUPP dams exhibited an impaired IPGTT, decreased circulatory insulin and weakened pancreatic insulin immunoreactivity. Compared to the male offspring of the sham group, the body mass of male RUPP offspring significantly caught up after PND42, but it was not sex-specific. RUPP pups also exhibited upregulations in glucagon (only males) and ghrelin (both sexes with a more significant increase in males) during PND14–PND60. However, in sham offspring (both sexes), glucagon levels were downregulated and ghrelin levels unchanged during PND14–PND60. The blood pressure, HOMA-IR and 24hUP values did not alter in RUPP pups.

**Conclusions:**

The overall results suggest that maternal RUPP has negative and sex-specific impacts on insulin, glucagon and ghrelin regulations in offspring and that, as young adults, male RUPP rats may be more prone to develop obesity and diabetes.

## Highlights

- Pancreatic insulin production was reduced in adult male offspring born to reduced uteroplacental perfusion (RUPP) rats.
- Offspring born to RUPP dams exhibited sex-specific alterations in insulin, ghrelin and glucagon changes.

## Background

The intrauterine environment can influence fetal growth and development. The balanced intrauterine homeostasis is crucial for the development and growth of the fetus [1]. However, several pathological conditions such as diabetes, hypertension, smoking, infections, and drugs can alter the optimal environment during pregnancy [2, 3].

Hypertensive disorders like preeclampsia are prevalent pregnancy complications, with high morbidity and mortality worldwide. Between 1990 and 2019, the global incidence of hypertensive disorders has increased by about 10.92% and complicated 6–11% of all pregnancies [4]. Preeclampsia is a multisystem pregnancy disorder in which several pathological processes trigger a common pathway leading to maternal vascular endothelial damage and high blood pressure [5]. Clinically, preeclampsia is defined as a new-onset of hypertension after the 20th gestational week accompanied by proteinuria or evidence of maternal organ failure (liver, kidney, and lung) with or without proteinuria [6]. Evidence suggests that preeclampsia threatens maternal health by increasing the risks of developing hypertension and metabolic diseases and can also affect the fetus’s health in later life [7].

Our knowledge about preeclampsia’s metabolic effects and their related mechanisms in offspring is based on a few epidemiological studies with a limited sample size [8]. Although some studies have reported that offspring born to preeclamptic mothers are at risk for overweight and metabolic disorders in adulthood, the exact mechanisms remain unclear [7]. Recent studies suggest that fetuses adapt to the intrauterine stressors during pregnancy through a concept called developmental programming, fetal programming, and developmental plasticity. Fetal programming can increase the risk of chronic diseases later in life [9]. A recent animal study revealed that the impairment of uterine perfusion in rats during pregnancy caused a mild cardiometabolic adaptation, notably a 51% reduction in fetal myocardial glucose levels on gestational day 19 [10]. These findings highlight the important areas for future research with long-term perspectives to investigate the underlying mechanisms. Evidence also indicates that gastroenteropancreatic peptides play essential roles in fetal programming, growth and development [11]. Ghrelin is a 28-amino-acid peptide that participates in fetal programming, growth and development of humans and rodents [12–15]. It has been reported that dysregulations in ghrelin signaling are associated with developmental disorders [16]. Like ghrelin, the pancreatic hormone glucagon plays an important role in the developmental programming of late-onset metabolic diseases such as insulin resistance and type-2 diabetes [17].

This study aimed to investigate the impact of the reduced uteroplacental perfusion (RUPP) rat preeclampsia model on offspring's metabolic outcomes.

## Methods

### Animals and breeding

Wistar rats (8 weeks old) were obtained from Birjand University of Medical Sciences’ Animal House (Birjand, Iran). Animals were housed in standard plastic cages under a controlled environment (12-h light/dark cycles and 22–24 °C temperature) and fed with a standard laboratory animal's pelleted diet (Behparvar Co., Iran) and tap water, ad libitum. In compliance with the ARRIVE guidelines, the Ethics Committee of the Birjand University of Medical Sciences approved the experimental procedures (permit code: Ir.bums.REC.1399.156).

**Fig. 1:**
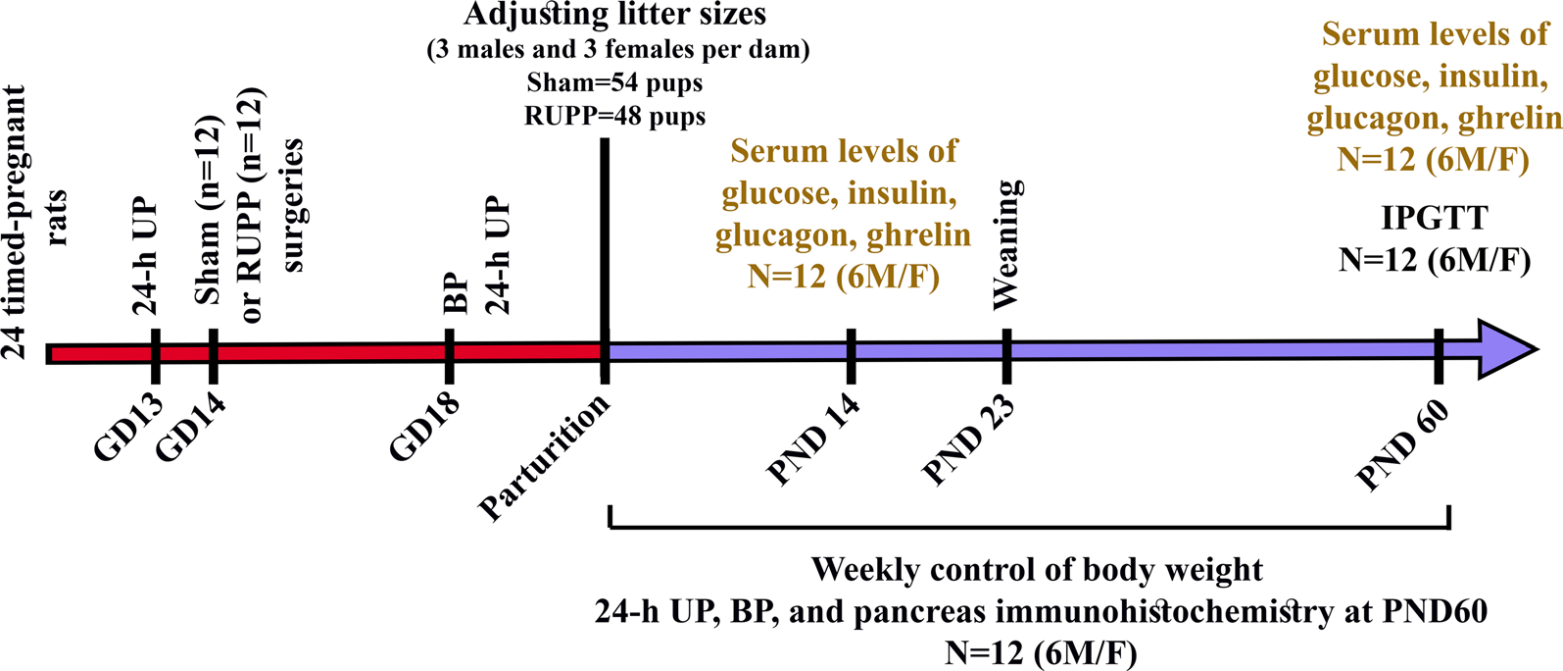
Experimental design diagram. *GD* gestational day, *BP* blood pressure, *24-h UP* 24-h urine protein excretion, *PND* postnatal day, *IPGTT* intraperitoneal glucose tolerance test

Female rats (*n* = 30) were mated with age-matched males (3:1). One day after the breeding, the presence of sperms in vaginal smears was considered as gestational day zero (GD0). Following pregnancy confirmation, 24 timed-pregnant rats were randomly allocated into two equal groups (Sham and RUPP). A schematic drawing of the experimental design is shown in Fig. 1.

### RUPP procedure

The RUPP was induced via arterial stenosis (abdominal aorta and each of the uterine artery branches) on GD14 as described previously [18]. In brief, timed-pregnant rats (GD14) were anesthetized using isoflurane (5% for induction and 2.5% for maintenance with 100% O_2_ as carrier) [19]. After anesthesia induction, each rat was placed on a controlled heating pad (37 °C; 709-0250; Narco BioSystems, Austin, USA), the abdominal fur was shaved, and the surgical site was disinfected with 70% ethanol (first) and povidone-iodine (2-time). The abdominal cavity was opened via a midline incision (3–4 cm length), the lower abdominal aorta was gently separated from the inferior vena cava and lifted with a 4–0 silk suture. Then, a sterile silver coil (0.203 mm internal diameter; 2.5 mm length) was wrapped around the aorta above the iliac bifurcation. In addition, branches of the right and left ovarian arteries that supply the uterus were constricted directly before the first segmental artery using sterile silver coils (0.100 mm internal diameter; 2 mm length) to prevent compensatory blood flow from the uterine branches of ovarian arteries. The inner diameters of the micro-coils were optimized to reduce uterine perfusion pressure by around 40% [20]. The sham group underwent the same procedure as the RUPP group but without micro-coils placement. After the surgery, a single dose (2 mg/kg) of the bupivacaine:lidocaine mixture (1:1, 0.2 mL) was given subcutaneously as an analgesic in both groups for 3 days [21]. Pregnant rats were housed singly before and after surgeries and were fed with standard rodent chow and tap water. All animals were acclimatized to metabolic cages for 2 days before urine collection. They were placed in acclimation chambers similar to metabolic cages with clean bedding.

### Measurement of maternal arterial blood pressure

On the GD18, three rats from each group were randomly selected and anesthetized by intraperitoneal (i.p.) injection of a hypnotic dose of sodium pentobarbital (45 mg/kg) [21]. In order to confirm the induction of preeclampsia in animals, the blood pressure was measured via carotid arterial catheterization as previously described [22]. The anesthetized rats were placed on a controlled heating pad (37 °C), and their right common carotid artery was gently exposed. Then, a fine micro-tip catheter (internal diameter: 0.5 mm) pre-filled with heparinized saline solution (0.5 IU/mL) was inserted into the common carotid artery toward the heart. The catheter was fixed by tiding a thread without obstructing the blood flow in the carotid cannula. In order to record blood pressure, the cannula was connected to a calibrated pressure transducer (SP844; Capto, Skoppum, Norway) coupled to an amplifier (Bridge Amp, ML221; AD Instruments, Bella Vista, NSW, Australia) and an acquisition system (Power Lab 4/30, ML866; AD Instruments). All the data were viewed and recorded by Lab Chart 7 computer software. All recordings were carried out for a period lasting 25–30 min. The used animals for blood pressure measurement were excluded from the study.

### Measurement of 24-h urinary protein (24hUP) excretion in pregnant rats

Pregnant rats of sham and RUPP groups were placed separately in metabolic cages on GD13 (before surgery) and GD18, and their 24-h urine samples were collected. After measuring the total 24-h urine volume, total protein content was determined with Pars Azmun’s total urine protein kit (Karaj, Iran).

### Bodyweight and metabolic profile of offspring

All pregnant rats were allowed to deliver, and the day of birth was defined as postnatal day 1 (PND1). Immediately after parturition, litter size, survival, sex and body weight of offspring were recorded. Then, the number and sex of all offspring were equalized to six pups per dam (three males and three females) to ensure equal nutrition access for all pups. Accordingly, nine mothers of sham (54 pups) and eight mothers of the RUPP (48 pups) groups were included in the study.

On the PND14 and PND60, 12 pups (six males/females) from each group were randomly selected (from different mothers), anesthetized, and their blood samples were collected. The blood samples were centrifuged at 2500 g for 15 min, and the sera were collected and stored at − 70 °C until analyses. In the serum samples, ELISA assay kits were used to determine biochemical parameters, including glucose (LOT: 1399-8, Pars Azmun, Iran), insulin (CK-E30620, Hangzhou Eastbiopharm, China), ghrelin (KA1863, Abnova, Taiwan) and glucagon (CK-E92165, Hangzhou Eastbiopharm, China). In addition, on the PND60, another 12 rats (six males/females) were placed in metabolic cages to collect 24-h urine samples. Then, their blood pressure was recorded, as mentioned above, and their pancreatic tissues were harvested for immunehistochemical investigations.

### Intraperitoneal glucose tolerance test (IPGTT)

In order to determine changes in insulin sensitivity, an i.p. glucose tolerance test (IPGTT) was performed at PND60 as previously described [23]. Briefly, 12 other randomly selected rats (six males/females) from each group were fasted overnight (15 h). Before starting the glucose challenge, basal glucose and insulin concentration (time 0) were determined via lancing the tail vein. Then, all animals received a single i.p. dose of 2 g/kg glucose, and their blood samples were taken at 30, 60, 90 and 120 min after glucose administration from the tail vein and glucose concentrations were determined with a glucometer (Accu-Chek^®^ Active, Roche Diagnostics GmbH, Mannheim, Germany). The glucose tolerance curve area (AUC) was calculated over the 120-min sampling period [24]. Based on the results of fasting glucose and insulin concentrations, the Homeostatic Model Assessment for Insulin Resistance (HOMA-IR) was calculated as follows: [glucose (mmol/l) × fasting insulin (mmol/l)]/ 22.5 [25].

### Pancreas morphology and immunohistochemistry

The harvested pancreatic tissues were fixed in 4% paraformaldehyde solution for 48 h. The fixed samples were embedded in paraffin wax and sectioned at 5 μm thickness. Then, the slides were deparaffinized and rinsed in Tris-buffered saline (TBS). The antigen retrieval step was performed by boiling the slides in sodium citrate buffer (10 mM sodium citrate, 0.05% Tween 20, pH 6.0) for 20 min. Next, the slides were cooled by placing the staining jar in running tap water for 10 min. Slides were washed two times with TBST (TBS plus 0.025% Triton X100) and blocked in 10% normal goat serum with 1% bovine serum albumin (BSA) in TBS for 2 h at room temperature. Subsequently, sections were incubated with a rabbit polyclonal anti-insulin primary antibody (ab63820, ABCAM) diluted (1:100) in TBS containing 1% BSA for 12 h at 4 °C. After that, slides were washed twice with TBST and immersed in freshly prepared 0.3% H2O2 in TBS for 15 min to block the endogenous peroxidase activity. The slides were washed (TBST) again and incubated with the secondary antibody (goat anti-rabbit IgG-HRP, 1:300, ab7090, ABCAM) for 1 h at room temperature. The slides were rinsed three times in TBS, and signals were visualized using 3,3′-diaminobenzidine (0.03%) for 10 min at room temperature. Finally, sections were counterstained with hematoxylin, dehydrated, cleared, and mounted. Slides were evaluated under a light microscope (Euromex-CMEX-10, Netherlands), and high-resolution images were taken using a digital camera (Image Focus v2, Netherlands). The immunohistochemical quantification of images was performed and represented as positive immunoreactive areas per islet area (%) as previously described [26]. In addition, the intensity of positive immunoreactivity was quantified by measuring optical density (OD) with the IHC Profiler plugin in Image J (1.48, NIH, USA). All the images were deconvoluted, and the insulin intensity was measured in the deconvoluted DAB images [27]. Based on the results of the pixel intensity values (the percentages of strong positive, positive, low, and negative intensities), the OD was calculated according to the equation:

OD = [(% contribution of strong positive × 4) + (% contribution of positive × 3) + (% contribution of low positive × 2) + (% contribution of negative × 1)]/100 [28].

### Statistical analysis

All values are reported as the mean ± SD.; *n* denotes the number of animals examined. Analyses were performed using GraphPad Prism (version 8.4.3) software (GraphPad Software Inc., San Diego, CA, USA) and SAS software (version 9.2, SAS Institute, Cary, NC). Statistical significance was determined with Student’s *t*-test, or two-way analysis of variance (ANOVA) for variables of the group (sham vs RUPP) and sex (male vs female). Tukey’s test (when the variances were equal) or Games–Howell analysis (when the variances were different) was performed for post hoc comparisons. Repeated-measures ANOVA or generalized estimating equation was used in timewise comparisons. Statistical significance was considered for *p* values < 0.05.

## Results

### Maternal effects of RUPP

Table 1 shows the maternal effects of RUPP in rats.

**Table 1:** Maternal parameters and reproductive outcomes of sham and RUPP (preeclampsia) groups

Parameter	Sham	RUPP	*p*-value
Maternal body weight at GD13 (before surgery)	239.5 ± 6.81	237.1 ± 7.30	0.49
Maternal body weight at GD20	308.3 ± 16.20	284.3 ± 12.39	0.001
GD13 urine protein exertion (mg/24 h)	8.25 ± 0.52	7.91 ± 0.73	0.38
GD18 urine protein exertion (mg/24 h)	8.08 ± 0.80	14.33 ± 1.50	< 0.001
GD18 blood pressure (mm/Hg)	86.66 ± 9.86	155.66 ± 11.59	0.001
GD18 heart rate (bits/minute)	241.33 ± 10.26	253.00 ± 32.07	0.58
Number of fetuses at surgery (GD14)	11.58 ± 1.16	12.8 ± 1.50	0.38
Litter size at birth	10.66 ± 1.50	11.00 ± 2.28	0.77
Number of females per litter	5.83 ± 2.22	5.66 ± 2.25	0.90
Number of males per litter	4.83 ± 1.32	5.50 ± 2.28	0.54
The mean percentage of the stillbirths	(3) 3.03%	(9) 8.71%	0.042

Values are presented as mean ± SD. RUPP: reduced uteroplacental perfusion, GD13: gestational day 13, GD18: gestational day 18. Maternal data are based on 12 pregnant rats per group, except for GD18 blood pressure (*n* = 3)

No significant differences in maternal body weight (*p* = 0.49) and 24hUP (*p* = 0.38) were found between the sham and RUPP pregnant rats before the surgical procedures (GD13). Four days (GD18) after the RUPP or sham surgeries, BP and 24hUP were significantly elevated in RUPP rats compared to the sham group (*p* = 0.001 and *p* < 0.001, respectively). At GD20, the maternal body weight of RUPP rats was significantly lower than sham-operated animals (*p* = 0.001).

At the time of surgical procedures (GD14), no significant difference was found in the number of fetuses between the sham and RUPP groups. After parturition, assessment of litter size and the number of male and female pups revealed no significant differences between sham and RUPP groups. However, the mean percentage of stillbirth ratio was significantly higher in the RUPP group than in the sham group (8.71% vs 3.03%, *p* = 0.042).

### Effects on offspring weight gain

**Fig. 2:**
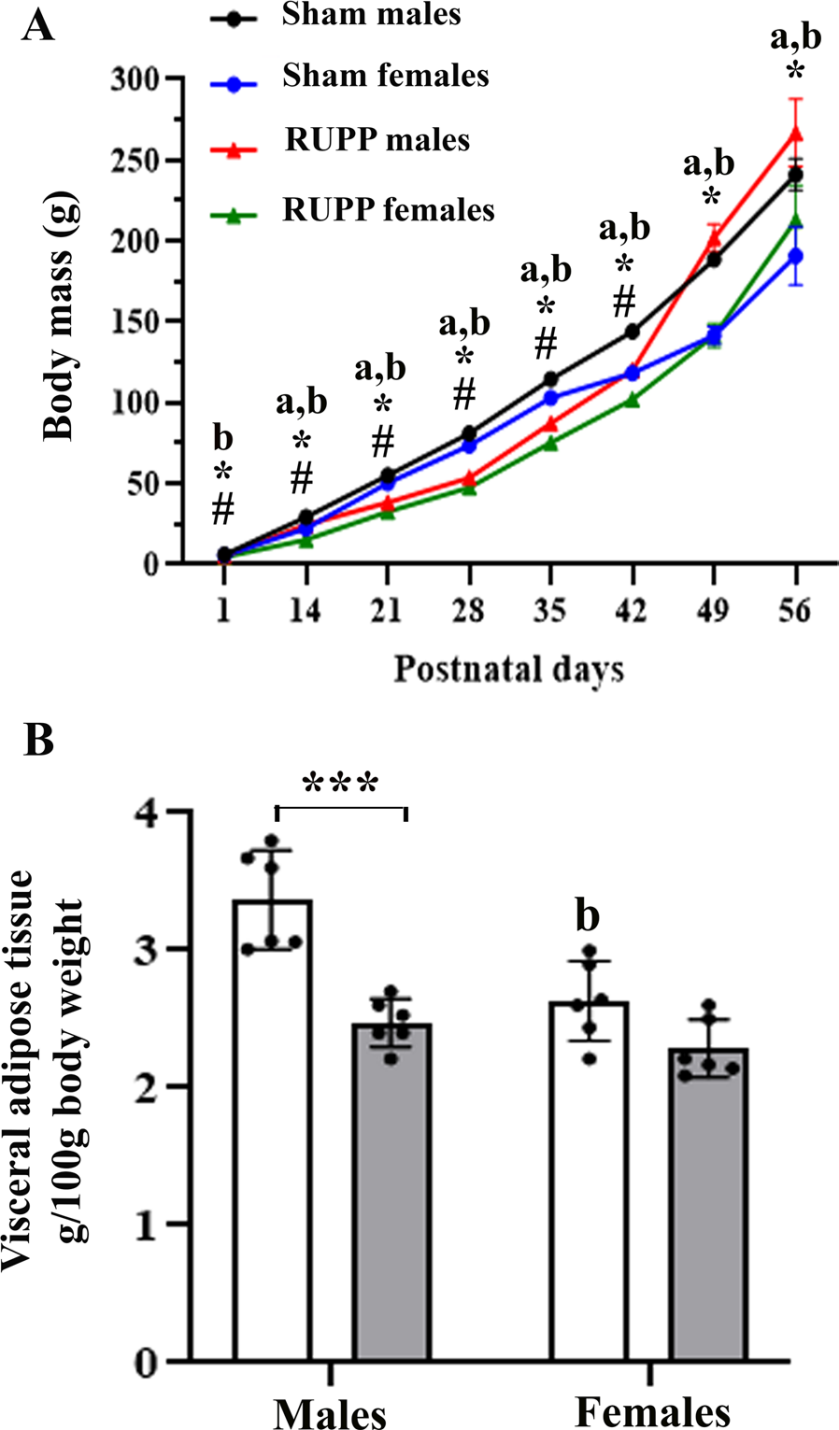
Effects of maternal reduced uteroplacental perfusion (RUPP) on offspring body mass growth (**A**) and visceral fat mass (**B**). Over 56 postnatal days, all pups gained weight. In both groups, males weighed more than females. The body mass of RUPP pups (both sexes) was significantly lower than their sex-matched peers in the sham group from birth until postnatal day 42. Male offspring of RUPP dams were heavier than male offspring of the sham group at postnatal days 49 and 56, but the effect was not a sex difference (group × sex interaction: *p* > 0.05). At postnatal day 60, male rats of the sham group showed a higher visceral fat mass than females, but in RUPP offspring, no difference was found by sex. Male offspring of the sham group had a higher visceral fat index than males of the RUPP group. Results are expressed as mean ± SD. (*n* = 6). The body mass data were analyzed by a repeated measure three-way ANOVA (time, sex, group) and Tukey’s post hoc test. *p < 0.05, RUPP males vs. Sham males and ^#^*p* < 0.05, RUPP females vs. Sham females. The data of PND60 visceral fat mass index were analyzed by two-way ANOVA (sex and group) and Tukey’s post hoc test: ***p < 0.001; (**a**) main effect of time; (**b**) the main effect of sex

The body weight of newborns was measured weekly from PND1 to PND56 (Fig. 2A). A three-way repeated measure ANOVA found that the main effects of group [*F*(1,160) = 381.2, *p* < 0.001], sex [*F*(1,160) = 67.54, *p* < 0.001] and time [*F*(7,160) = 27.48, *p* < 0.001] on body weight were all significant. Accordingly, the body weight of rat pups increased with age, and male rats were heavier than females. RUPP pups had a lower weight from PND1–PND42 than sham offspring. There were significant interactions between time × sex [*F*(7,160) = 37.95, *p* < 0.001], with females weighing less than males, and time × group [*F*(7,160) = 53.35, *p* < 0.001], with RUPP rats of both sexes had a lower body mass than their peers in the sham group from PND1 until PND42. Specific pairwise comparison showed that only male offspring of RUPP dams had higher body weight than male pups of the sham group at PND49 (*p* = 0.047) and PND56 (*p* = 0.016). However, it was not statistically sex-specific, because sex × group [*F*(1,160) = 0.54, *p* = 0.46] and time × sex × group [*F*(7,160) = 1.04, *p* = 0.404] interactions were not significant.

In addition, the data of visceral fat mass measured at PND60 were analyzed by two-way ANOVA. The main effect of the group was significant [*F*(1, 20) = 29.35, *p* < 0.001], with RUPP rats having lower visceral fat mass than sham rats (Fig. 2B). The main effect of sex was also significant [*F*(1, 20) = 16.12, *p* < 0.001], with males having more visceral fat mass than females. The group × sex interaction was also significant [*F*(1, 20) = 5.509, *p* = 0.029]. Male rats in the RUPP group had lower visceral fat mass than male rats in the sham group (*p* < 0.001), whereas no similar difference was found in female rats (*p* = 0.16).

### Effects on blood glucose and insulin

**Fig. 3:**
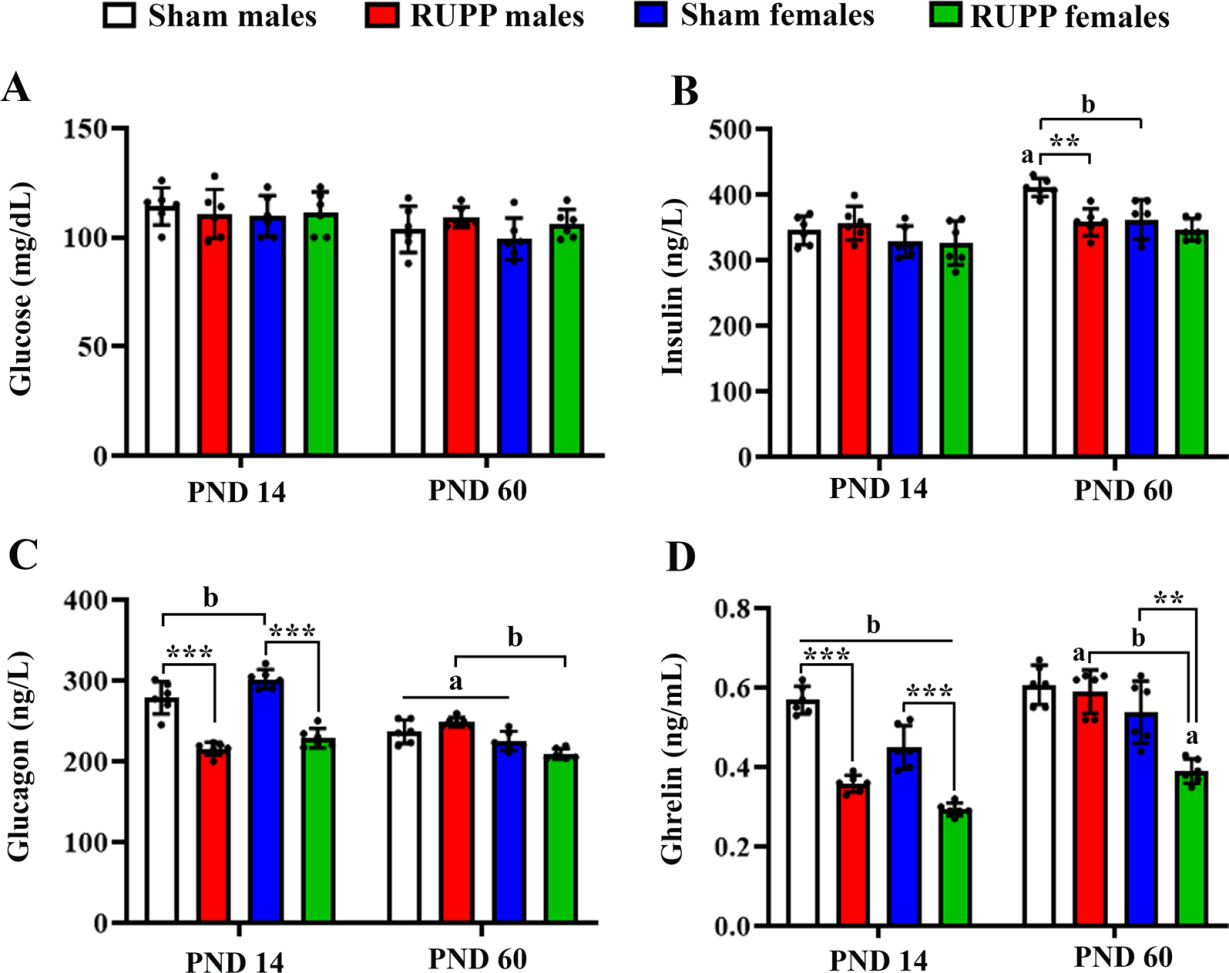
Effects of maternal reduced uteroplacental perfusion (RUPP) on offspring blood glucose (**A**), insulin (**B**), glucagon (**C**) and ghrelin (**D**) on postnatal days 14 and 60 (PND14 and PND60). In the male offspring of the sham group, insulin levels were upregulated from PND14–PND60, but insulin levels of the male offspring of RUPP dams did not change. At PND14, only female pups in the sham group had higher glucagon levels than males, and RUPP pups exhibited lower glucagon levels than their sex-matched counterparts in the sham group. Over time glucagon levels of sham offspring (both sex) were decreased, while in RUPP rats (only males), circulatory glucagon was increased. At PND60, male offspring of the RUPP group had higher glucagon levels than females. At PND14, male pups (both groups) exhibited higher ghrelin levels than females, but at PND60, only male rats of the RUPP group had higher ghrelin than females. RUPP pups (both sexes) had lower ghrelin than their peers in the sham group at PND14, but only female rats in the RUPP group showed lower ghrelin levels than the sham group at PND60. Over time, ghrelin levels were unchanged in the sham group, but its levels were upregulated in RUPP pups (both sex). Results are expressed as mean ± SD. (*n* = 6). Tukey’s post hoc test: **p < 0.01;***p < 0.001; (**a**) main effect of time and (**b**) main effect of sex

Biochemical and hormonal parameters including circulatory glucose, insulin, glucagon and ghrelin were measured at PND14 and PND60. No differences were observed between the studied groups on blood glucose at different ages [*F*(1, 5) = 1.44, *p* = 0.28] or by sex [*F*(1, 5) = 2/92, *p* = 0.068] (Fig. 3A).

At PND14, no differences were observed by group [*F*(1, 5) = 0.9366, *p* = 0.377] and sex [*F*(1, 5) = 3.599, *p* = 0.116] in circulatory insulin concentrations. However, at PND60, there was a statistically significant interaction between the effects of group and sex on circulatory insulin levels [*F*(1, 5) = 12.23, *p* = 0.017], with sham males having higher insulin levels than RUPP males (*p* = 0.005), RUPP females (*p* = 0.002), and sham females (*p* = 0.007). Assessment of insulin change over time (PND14–PND60) showed that only male offspring from the sham group displayed a significant change (*p* = 0.0008) (Fig. 3B).

### Effects on circulatory glucagon and ghrelin

At PND14, significant effects of the group [*F*(1, 5) = 148.3, *p* < 0.001] and sex [*F*(1, 5) = 13.08, *p* = 0.015] were found on plasma glucagon levels. RUPP offspring had lower glucagon levels than the sham group, and males had lower glucagon levels than females (Fig. 3C). However, the group × sex interaction was not significant [*F*(1, 5) = 0.9834, *p* = 0.366]. At PND60, female rats exhibited lower glucagon levels [*F*(1, 5) = 28.27, *p* = 0.003] than males and no difference was observed between the studied groups [*F*(1, 5) = 0.2522, *p* = 0.633]. The group × sex interaction was not significant [*F*(1, 5) = 1.553, *p* = 0.267]. Assessment of glucagon change over time (PND14–PND60) showed that glucagon levels in the sham group (both sexes) were significantly downregulated at PND60 (*p* = 0.005 in males and *p* < 0.001 in females). Conversely, male offspring from RUPP dams exhibited an elevated trend of circulatory glucagon (*p* = 0.003). No significant glucagon change was observed in female RUPP pups from PND14–PND60 (*p* = 0.13).

At PND14, significant effects of group [*F*(1, 5) = 163.1, *p* < 0.001] and sex [*F*(1, 5) = 32.18, *p* = 0.0024] were found on circulatory ghrelin levels (Fig. 3D). Females had lower ghrelin levels than males, and RUPP offspring exhibited lower ghrelin levels than the sham group. The interaction of group × sex was not significant [*F*(1, 5) = 6.305, *p* = 0.053]. At PND60, there was a statistically significant interaction between the effects of group and sex on circulatory ghrelin levels [*F*(1, 5) = 20.73, *p* = 0.006], with RUPP females having lower ghrelin levels than sham females (*p* = 0.003), RUPP males (*p* = 0.0007), and sham males (*p* = 0.0005). Assessment of ghrelin change over time (PND14–PND60) showed that ghrelin levels were not changed in both male (*p* = 0.56) and female (*p* = 0.42) rats of the sham group. On the other hand, ghrelin levels were significantly upregulated in both males (*t* = 8.95, *p* = 0.001) and females (*t* = 10.13, *p* = 0.0006) of the RUPP group.

### Effects on HOMA-IR and IPGTT

**Fig. 4:**
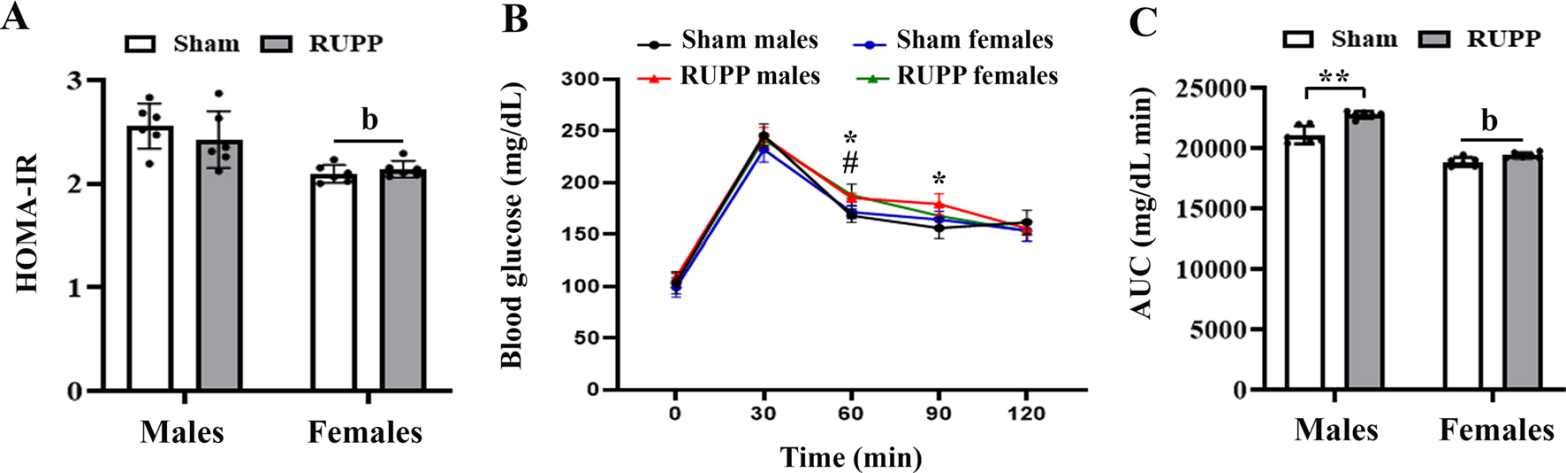
Effects of maternal reduced uteroplacental perfusion (RUPP) on offspring HOMA-IR (**A**), intraperitoneal glucose tolerance test (IPGTT) (**B**) and glucose clearance rate, as reflected by the area under IPGTT curves (**C**) on postnatal days 60. Male rats in the RUPP group exhibited a slower glucose clearance than male rats in the sham group. Results are expressed as mean ± SD. (*n* = 6). Repeated-measure ANOVA followed by Sidak’s post hoc test was used to analyze IPGTT data. In IPGTT curves (B): *p < 0.05, RUPP males vs. Sham males; ^#^*p* < 0.05, RUPP females vs. Sham females. (**b**) show a significant main effect of sex. **p < 0.01

At PND60, HOMA-IR was varied according to sex [*F*(1, 5) = 22.51, *p* = 0.005], with females showed lower HOMA-IR than males (Fig. 4A). However, no difference was observed between the studied groups [*F*(1, 5) = 0.4605, *p* = 0.52] and the interaction of sex × group was not significant [*F*(1, 5) = 3.723, *p* = 0.11].

At PND60, the baseline blood glucose levels prior to IPGTT were comparable across the studied groups [*F*(3, 20) = 1.52, *p* = 0.23]. However, glucose concentration during the IPGTT varied significantly with time [*F*(3.376, 67.53) = 791.3, *p* < 0.001] and group [*F*(3, 20) = 3.626, *p* = 0.0308]. In addition, interaction of time × group was significant [*F*(12, 80) = 2.903, *p* = 0.002] (Fig. 4B). Significant differences of glucose levels between the studied groups were found at 60 min [*F*(1, 5) = 14.27, *p* = 0.0129] and 90 min [*F*(1, 5) = 9.25, *p* = 0.028] following 2 g/kg glucose administration. Sixty minutes after the glucose loading, both male and female rats of the RUPP group had higher glucose levels than their sex-matched peers in the sham group (*p* = 0.012, and *p* = 0.016, respectively). However, at the studied time point (60 min), the main effect of sex [*F*(1, 5) = 2.87, *p* = 0.15] and sex × group interaction [*F*(1, 5) = 0.04, *p* = 0.83] were not significant. At 90 min after the glucose administration, only male RUPP rats had significantly higher glucose concentrations than sham males [group × sex interaction: *F*(1, 5) = 11.14, *p* = 0.020].

The overall change in blood glucose levels during 120 min following glucose administration was measured as AUC (Fig. 4C). The AUC was varied according to sex [*F*(1, 5) = 97.74, *p* = 0.0002] and group [*F*(1, 5) = 69.46, *p* = 0.0004]. Females had lower glucose AUC than males, and the RUPP group had higher AUC than the sham group. The interaction of sex × group was also significant [*F*(1, 5) = 6.76, *p* = 0.048], with male rats of the RUPP group exhibiting a slower glucose clearance than male rats of the sham group (*p* = 0.010).

### Effects on pancreatic morphology and insulin secretion

**Fig. 5:**
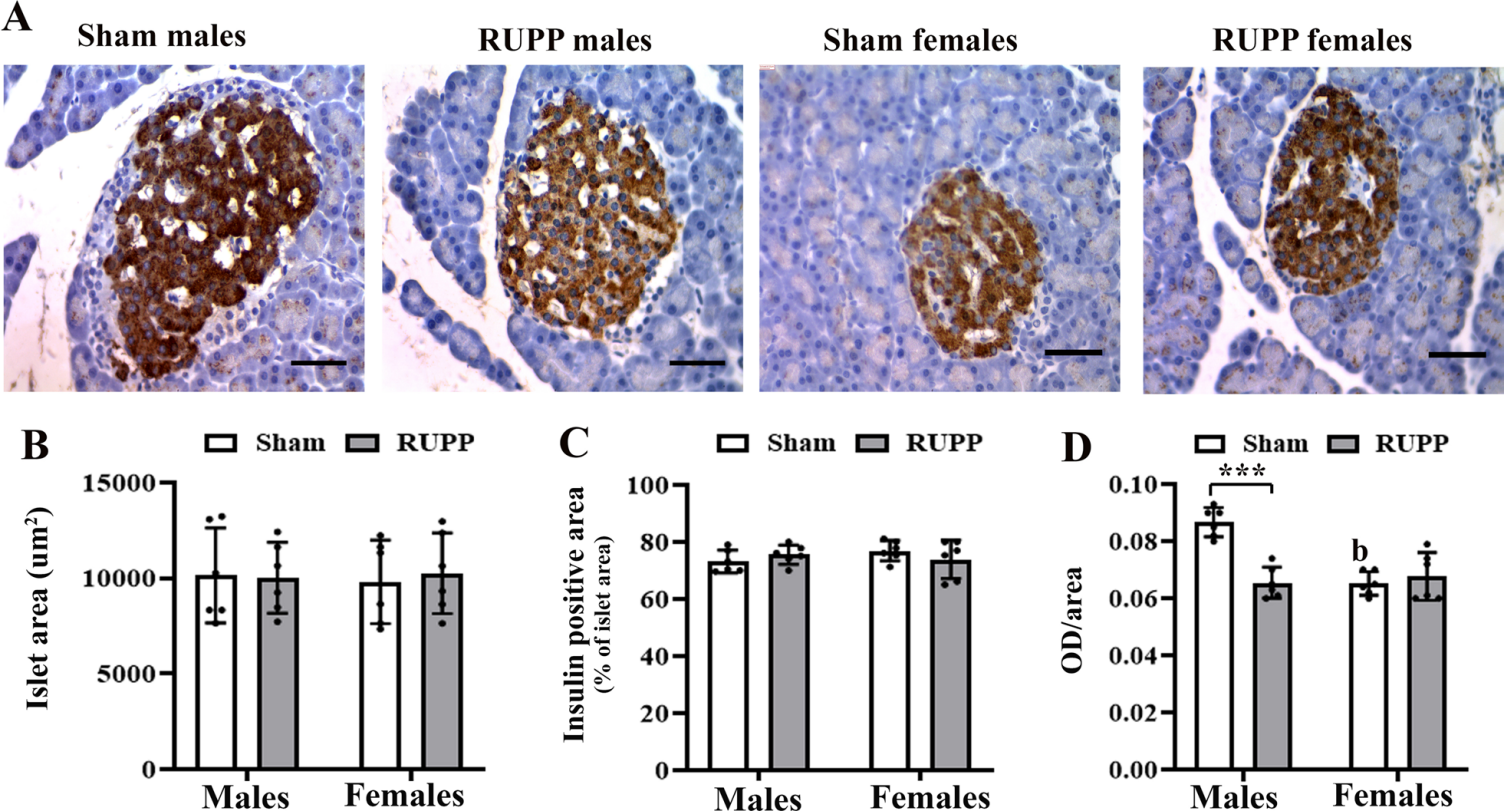
Effects of maternal reduced uteroplacental perfusion (RUPP) on offspring pancreatic islets insulin immunohistochemistry (**A**) at the age of 2 months. Islet area (**B**), insulin-positive area per total islet area (**C**), and quantification of insulin reactivity by optical density per islet surface area (**D**). Male rats in the RUPP group exhibited lower insulin intensity than male rats in the sham group. Bars represent the mean ± SD for each group, *n* = 6. Two-way ANOVA and post hoc Tukey’s multiple comparisons: **p < 0.01; (**b**) main effect of sex

No differences were observed by sex [*F*(1, 5) = 0.004, *p* = 0.94] and group [*F*(1, 5) = 0.09, *p* = 0.77] in surface area of pancreatic islets and also in the positive immunoreactive area per islet area [*F*_sex_(1,5) = 0.209, *p* = 0.66; *F*_group_(1,4) = 0.05, *p* = 0.83] (Fig. 5A–C). However, There was an interaction between sex × group [*F*(1, 5) = 20.86, *p* = 0.006] on the intensity of insulin immunoreactivity. Male rats in the RUPP group exhibited lower insulin intensity than their peers in the sham group (*p* = 0.005) (Fig. 5D).

### Effects on offspring blood pressure and 24hUP

**Fig. 6:**
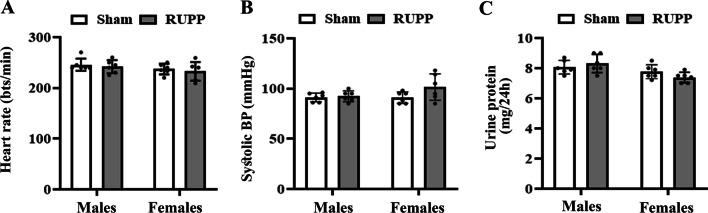
Effects of maternal reduced uteroplacental perfusion (RUPP) on offspring heart rate (**A**), systolic blood pressure (**B**), and 24-h urine protein excretion (**C**) at the age of 2 months. No significant differences were found by group and sex. Bars represent the mean ± SD for each group, *n* = 6. Data were analyzed by two-way ANOVA

At PND60, no significant differences were observed in heart rate [*F*_sex_(1,5) = 2.94, *p* = 0.14; *F*_group_(1,5) = 1.02, *p* = 0.35], systolic blood pressure [*F*_sex_(1,5) = 0.62, *p* = 0.46; *F*_group_(1,5) = 3.12, *p* = 0.13], and 24hUP [*F*_sex_(1,5) = 6.40, *p* = 0.052; *F*_group_(1,5) = 0.71, *p* = 0.43] (Fig. 6A–C) between the studied groups.

## Discussion

As reported by other investigators, a rat model of RUPP was successfully established as confirmed by elevated maternal blood pressure and proteinuria [29–31]. It is well-known that animal models could help us distinguish the independent and/or dependent role of each pathophysiological change during preeclampsia and after birth [32]. The RUPP models have been extensively used in rodents and have shown various markers compatible with the human phenotype, including oxidative stress, hypertension, and antiangiogenic profile [29].

Several studies demonstrated that the incidence of low birth weight in preeclampsia-affected pregnancies is higher than in non-preeclampsia [33, 34]. In the present study, RUPP offspring (both sexes) exhibited lower body mass than sham pups during PND1–PND42. However, male pups from the RUPP group exhibited significantly higher body weight than those from the sham group at PND49 and PND60. These findings recapitulate earlier findings indicating that offspring affected by preeclampsia may have an increased risk of obesity in adulthood [35]. It has been previously reported that pups born after undernourishment conditions such as intrauterine growth restriction (IUGR) and preeclampsia tend to compensate for their growth restriction in the postnatal period [14, 35]. Although the exact mechanisms underlying this adaptation are still unclear, the ghrelin hormone has been proposed to be involved in early infancy weight gain. A report indicates that higher serum levels of ghrelin are associated with faster postnatal catch-up growth [36]. In the present study, offspring from the RUPP group (mainly males) showed a significant upregulation in ghrelin levels from PND14–PND60.

In contrast, the ghrelin levels in the sham group were downregulated during the studied time. Recent studies demonstrated that ghrelin signaling is dysregulated in offspring from complicated pregnancies such as preeclampsia and gestational diabetes [37, 38]. We previously have reported that plasma ghrelin levels in male offspring born to diabetic rats were significantly altered at PND14 [39]. In addition, it has been recently demonstrated that ghrelin signaling was dysregulated in male rat offspring with growth restriction and IUGR, but did not change in female pups [40].

We also found that circulatory glucagon levels were significantly decreased in sham offspring (both sexes) from PND14–PND60. Conversely, male offspring from RUPP dams exhibited an upregulation in circulatory glucagon during the period. It has been demonstrated that glucagon stimulates ghrelin secretion, and there is a positive feedback loop between ghrelin secretion and glucagon levels [41]. Male offspring from RUPP dams at PND60 exhibited significantly lower circulatory insulin concentrations concomitant with a reduction in pancreatic insulin contents and glucose intolerance than the male offspring of the sham group. Recently, Akhaphong et al., in an elegant study, evaluated the insulin production in offspring from RUPP dams at embryonic day 19.5. They found that circulating insulin level was not altered in either male or female RUPP fetuses at embryonic day 19.5. However, the fetuses showed (both sexes) insignificant (*p* = 0.064 for males and *p* = 0.08 for females) lower pancreatic insulin contents than their peers in the sham group. They have concluded that the event may enhance the susceptibility of RUPP offspring to type-2 diabetes in adulthood, and further study could look at the insulin secretion in adult RUPP rats [42]. The insulin reduction observed in RUPP offspring (male pups) could partially explain glucose intolerance. In addition, the elevation of ghrelin might be one of the possible causes of suppressing insulin elevation and glucose intolerance in male RUPP rats. It has been previously reported that ghrelin could inhibit insulin secretion from pancreatic islets [43]. A randomized, double-blind placebo-controlled crossover study showed that constant intravenous ghrelin infusion in healthy young men significantly reduced insulin sensitivity [44].

There was no significant difference in HOMA-IR score between the studied groups. HOMA-IR is a simple method suggested as a surrogate marker for insulin resistance. However, its advantages in animal studies are notorious. The insulin resistance test is a valid and more reproducible test for assessing insulin sensitivity in experimental animals [45]. To our knowledge, no study has investigated insulin sensitivity in adult offspring born to preeclamptic mothers. It has been previously suggested that IUGR is associated with an increased risk of developing insulin resistance in adulthood, but the underlying mechanisms remain unclear. Interestingly, Xing and coworkers found that IUGR pups with rapid postnatal catch-up growth (similar to our study) exhibited insulin resistance in skeletal muscle tissues at 2 and 4 months after birth. Their mechanistic investigations revealed that the expression of glucose transporter type 4 in the skeletal muscles of IUGR offspring was downregulated through suppression of PI3K/Akt-related signaling activities [46].

In the present study, the blood pressure and urine protein in offspring (both sexes) born to RUPP dams did not alter in early adulthood. Although these parameters were associative data, our findings were distinct from other studies demonstrating that maternal preeclampsia is slightly associated with higher offspring blood pressure from early childhood to adolescence [47, 48]. However, our results support previous findings by Moritz and coworkers, showing no differences in blood pressure between offspring born to dams with vs without uteroplacental insufficiency perfusion at 8, 12 and 20 weeks of age [49]. However, in another previous study, offspring from the RUPP pregnant Sprague–Dawley rats exhibited significant elevations of mean arterial pressure as early as four weeks of age [50]. The inconsistencies might be attributed to the differences between the strains of rats employed in the mentioned studies. In the present experiment, as well as Moritz and coworkers’ study, Wistar rats were used. It has been previously reported that the predisposition to hypertension was higher in Sprague–Dawley rats than in Wistar or Fischer 344 rats of the same age [51]. Moreover, it has been previously suggested that early catch-up growth due to providing a normal lactational environment for rat pups born to dams that underwent bilateral uterine vessel ligation may protect against the onset of hypertension in their later life [52]. In the present study, the catch-up growth occurred after weaning, indicating that lactation might be associated with the reduced weight gain of RUPP offspring. Further studies are needed to test the impacts of cross-fostering between RUPP and normal dams on the growth and metabolic responses of RUPP offspring.

It is well established that cardiometabolic diseases usually emerge with age, while in the present study, RUPP offspring were evaluated until young adolescence (60 days). However, these findings are valuable in light of understanding that early alterations in pancreatic function and metabolic-related hormones might be associated with the development of metabolic diseases in the later life of offspring exposed to preeclampsia.

### Perspectives and significance

Our finding of a sex-specific metabolic response in the offspring born by rats with experimental preeclampsia is novel and suggests a possible mechanism for long-term metabolic consequences. These findings recapitulate earlier findings indicating that offspring affected by preeclampsia may have an increased risk of obesity and diabetes in adulthood [35]. We found that male offspring are more susceptible than females to diabetes risk in adulthood, possibly due to altered metabolic regulations of insulin, ghrelin, and glucagon during their early life. These findings will likely provide much-needed clinical insights, and further studies are needed to explore which factors contribute to the metabolic response.

## Conclusion

In summary, the results of the present study support previous hypotheses that an abnormal intrauterine milieu can induce glucose metabolism abnormalities and insulin resistance later in life. We have shown that maternal RUPP has negative and sex-specific impacts on the regulation of insulin, glucagon and ghrelin in offspring and that, as young adults, male rats may be more prone to develop diabetes.

## Data Availability

The datasets used and/or analyzed during the present study are available from the corresponding author on reasonable request.

## Abbreviations

RUPP: Reduced uteroplacental perfusion
PND: Postnatal day
IPGTT: Intraperitoneal glucose tolerance test
HOMA-IR: Homeostatic model assessment of insulin resistance
24hUP: 24-Hour urine protein
GD: Gestational day
i.p.: Intraperitoneal
AUC: Area under curve
TBS: Tris-buffered saline
TBST: Tris-buffered saline with Tween20
BSA: Bovine serum albumin
OD: Optical density
IUGR: Intrauterine growth restriction
